# Use of *Torulaspora delbrueckii* Co-fermentation With Two *Saccharomyces cerevisiae* Strains With Different Aromatic Characteristic to Improve the Diversity of Red Wine Aroma Profile

**DOI:** 10.3389/fmicb.2018.00606

**Published:** 2018-04-05

**Authors:** Bo-Qin Zhang, Yu Luan, Chang-Qing Duan, Guo-Liang Yan

**Affiliations:** ^1^Centre for Viticulture and Enology, College of Food Science and Nutritional Engineering, China Agricultural University, Beijing, China; ^2^Key Laboratory of Viticulture and Enology, Ministry of Agriculture, Beijing, China

**Keywords:** *Torulaspora delbrueckii*, *Saccharomyces cerevisiae*, mixed fermentation, simultaneous inoculation, sequential inoculation, diversified aroma profile

## Abstract

The use of selected *Saccharomyces* and non-*Saccharomyces* strains as mixed starters has advantages over pure fermentation due to achieving wine products with distinctive and diversified aroma expected by consumers. To obtain a way to improve the aroma diversity and increase the differentiation of wine product, in this study, the aromatic effect of multi-culture of indigenous *Torulaspora delbrueckii* (*TD*12), simultaneous and sequential inoculation with two *Saccharomyces* strains (indigenous icewine yeast *SC*45 and commercial yeast BDX) with different enological characteristics were investigated in laboratory-scale 20 L fermenter, respectively. The results showed that *T. delbrueckii* co-fermented with different *S. cerevisiae* strain could generate diversified physicochemical and aromatic quality of wine as evidenced by PCA. Mixed fermentation of *SC*45/*TD*12 produced higher contents of higher alcohol (3-methyl-1-pentanol and phenylethyl alcohol), ethyl esters (ethyl decanoate and ethyl butanoate), terpenes and phenylacetaldehyde with less fatty acids (hexanoic acid, octanoic acid) and acetic acid, while BDX/*TD*12 generated more C_6_ alcohol (1-hexanol) and acetate esters (ethyl acetate and isoamyl acetate). Compared to simultaneous inoculation, sequential inoculation could achieve higher aroma diversity, and generate higher intensity of fruity, flowery and sweet attributes of wine as assessed by calculating the odor activity values. The different *S. cerevisiae* strain and inoculation method in alcoholic fermentation could further influence the formations of aromatic compounds in malolactic fermentation. Our results highlighted the importance of *S. cerevisiae* strain in shaping the aromatic quality of wine in mixed fermentation, and also suggested that using different *S. cerevisiae* strains with distinct aromatic characteristics co-fermentation with specific non-*Saccharomyces* strain is a potential way to increase the aromatic diversity and quality of wine product, which could provide an alternative way to meet the requirement of wine consumers for diversified aromatic quality.

## Introduction

In recent years, the consumer requirements for distinctive aromatic characteristics urge winemakers to develop a variety of ways to manipulate specific aroma compounds and increase the complexity of wine. In this context, non-*Saccharomyces* yeasts have received more attention because these strains have desired enological characteristics that are absent in *S. cerevisiae*, such as producing high levels of aroma compounds and producing several enzymes (esterases, ß-glycosidases, lipases and proteases) (Andorrà et al., 2012; Jolly et al., 2014; Liu et al., 2016; Padilla et al., 2016). The multi-starter fermentations of different non-*Saccharomyces* species together with *S. cerevisiae* in a controlled manner can produce distinct aroma profiles and improve the complexity of final wine (Anfang et al., 2010; Imma et al., 2010; Cañas et al., 2011; Jolly et al., 2014).

Among these non-*Saccharomyces* yeast species, *Torulaspora delbrueckii* is probably the one that is most often used in wine-making. Co-fermentation with *S. cerevisiae* and this yeast has been described to improve wine complexity, decrease volatile acidity and acetaldehyde content, or increase dried fruit and pastry aromas (Bely et al., 2008; Renault et al., 2009; Azzolini et al., 2012, 2015; Velázquez et al., 2015). The mixed *T. delbrueckii/S. cerevisiae* inoculation can also increase the total ester concentration (such as isoamyl acetate, ethyl hexanoate and 3-hydroxybutanoate) compared to single inoculation with *T. delbrueckii* or *S. cerevisiae* (Herraiz et al., 1990). However, inconsistent results have also been reported. Comitini et al. (2011) and Sadoudi et al. (2012) showed that total concentration of ester in mixed inoculations was lower than that in pure *S. cerevisiae* culture, especially a significant reduction of isoamyl acetate. The main explanation for these contradictory results is the difference of *T. delbrueckii* strain used in these studies because the aromatic effect of non-*Saccharomyces* in the mixed fermentation is usually strain-dependent (Renault et al., 2009).

Until now, most works related to the mixed fermentation for improving the aromatic complexity are to combine different non-*Saccharomyces* species/strains with one specific *S. cerevisiae* strain; however, no detailed information are available on the aromatic impact of different *S. cerevisiae* strain with one non-*Saccharomyces* strain. Compared to non-*Saccharomyces* yeast species, *S. cerevisiae* have more diversified genetic characteristics and metabolic behaviors. Therefore, different *S. cerevisiae* strains can develop diversified aromatic profiles when fermenting with the same must (Marsit and Dequin, 2015; Eldarov et al., 2016). This can well explain why there are above 200 commercial *Saccharomyces* yeast strains available in the market. Due to the fact that different *S. cerevisiae* strains can generate different aromatic profiles, it is reasonably believed that one specific non-*Saccharomyces* strain paired with different *S. cerevisiae* strains with distinct enological characteristics could also produce the wine with diversified aromatic quality. Using autochthonous or locally selected wine yeast is encouraged in current wine-making because these yeasts can well adapt to the micro-conditions of the wine region and improve the aroma quality of wine by generating unique regional character (Calabretti et al., 2012; Liu et al., 2016). In our previous work, two potential wine yeast strains *S. cerevisiae* CVE-*SC*45 and *T. delbrueckii* CVE-*TD*12 were isolated from Dongbei province and Xinjiang province, two major wine regions in China, respectively. The preliminary experimental results showed that they have good technological characteristics, including high fermentation speed, low production of H_2_S, and high tolerance to ethanol, SO_2_ and sugar, and show good potential application in wine-making (Unpublished results). To enrich our understanding of beneficial effects of mixed fermentation on wine aroma quality, in this study, the effects of mixed fermentation of *T. delbrueckii* CVE-*TD*12 with two *S. cerevisiae* strains (indigenous icewine yeast CVE-*SC*45 and commercial yeast BDX with simultaneous and sequential inoculation) on aromatic profiles of wine were investigated in 20 L fermenter using Cabernet Sauvignon grape as the must, respectively. The cell growth, physicochemical products and aromatic compounds of wine after alcoholic fermentation and malolactic fermentation were determined and compared with pure *S. cerevisiae* fermentations, respectively.

## Materials and methods

### Yeast strains and grape must

Two indigenous wine yeast strains were used in this study, including *T. delbrueckii* CVE-*TD*12 and *S. cerevisiae* CVE-*SC*45. CVE-*TD*12 was isolated from spontaneous fermentative wines of Cabernet Sauvignon in Wujiaqu in the middle part of Xinjiang, China. CVE-*SC*45 was isolated from spontaneous fermentative icewine of Vidal Blanc in Huairen, Liaoning, China. They were identified by 5.8S-ITS and 26S rDNA-RELP analysis and deposited in China General Microbiological Culture Collection Center (CGMCC), and the preservation numbers are 12L-161017 (*T. delbrueckii* CVE-*TD*12) and 45-161017 (*S. cerevisiae* CVE-*SC*45). The commercial strain BDX (Lallemand S. A., Toulouse, France), which was usually used to ferment table red wine, was applied as another *Saccharomyces* strain. These strains were stored at −80°C in YPD medium with adding glycerol (20% v/v final concentration).

### Fermentation conditions and samples

Duplicated fermentations were conducted in Cabernet Sauvignon grape must. Eighteen kg of wine grapes from Changli, Hebei were added into laboratory-scale 20 L stainless steel fermenter after destemming, crushing and adding 60 mg/L of sulfur dioxide and 30 mg/l pectinase. The basic parameters of the original must were measured as follows: pH 3.16, 5.13 g/L of titratable acid, 226 g/L of reducing sugar. Mixed fermentation trails were performed by *TD*12 with *SC*45 and BDX simultaneous and sequential inoculation, respectively. Six trials were therefore set: (1) single inoculation with BDX; (2) Simultaneous inoculation of BDX and *TD*12 (SI-BDX/*TD*12); (3) sequential inoculation of *TD*12 followed by BDX after 2 days (SE-BDX/*TD*12); (4) single inoculation of *SC*45; (5) Simultaneous inoculation of *SC*45 and *TD*12 (SI-*SC*45/*TD*12); (6) sequential inoculation of *TD*12 followed by *SC*45 after 2 days (SE-*SC*45/*TD*12). These strains were pre-cultured in 500 mL shake flasks containing 300 mL of YPD medium overnight (30°C and 150 rpm). Cells were harvested by centrifugation and washed twice with sterile water. The inoculum ratio of non-*Saccharomyces* and *Saccharomyces* species was 10:1, and the initial active population of non-*Saccharomyces* and *Saccharomyces* were 1.0 × 10^7^ CFU/mL and 1.0 × 10^6^ CFU/mL, respectively (Renault et al., 2015). Fermentation proceeded for 7 or 9 days at 24–26°C with regular punching skins down to improve extraction. Five ml of fermenting musts were sampled each 24 h for counting yeast population. After alcoholic fermentation ended (sugar content was below 4 g/L), grape pomace was separated out of wine carefully. The wines were transferred to 10 L glass fermenter for settle for 2 days and start malolactic fermentation by inoculating commercial *Oenococcus oeni* (Viniflora® Oenos, Chr. Hanse) according the manufacturer's instructions. The samples after alcoholic fermentation and malolactic fermentation were drawn, centrifuged and stored at −20°C for analysis of glucose, fructose, main products (glycerol, acetic acid, ethanol and non-volatile acids) and volatile aroma compounds. Each sample was analyzed in triplicate.

### Analytical methods

The quantity of yeasts was determined by plating on WL nutrient agar containing 100 mg/L chloramphenicol inhibiting bacterial growth. To avoid any possible interference of non-*Saccharomyces* other than *T. delbrueckii*, the late-harvested Cabernet-Sauvignon grapes was used because the amount of non-*Saccharomyces* was very low in late-harvested grapes with adding 60 mg/L SO_2_ (Ramírez et al., 2016). Plates were incubated at 30°C for 72 h. On WL nutrient agar plates, yeast colonies belonging to *S. cerevisiae* and non-*Saccharomyces* were distinguished by their different morphological characteristics.

The amino acid concentration of original must was determined by high performance liquid chromatography (HPLC) method developed previously in our laboratory (Wang et al., 2014). The main products of final wines were determined by an HPX-87H Aminex ion-exchange column (300 × 7.8 mm, Bio-Rad Laboratories, Hercules, CA, USA) with 5 mM sulfuric acid as the mobile phase (Duan et al., 2015). The volatile compounds of final wines were determined by headspace solid-phase micro-extraction coupled with gas chromatography-mass spectrometry (HS-SPME-GC-MS) according to our previous study (Zhang et al., 2011; Xu et al., 2015; Liu et al., 2016). Analyses were performed in triplicate. The detailed quantitation information about quantitative ion, quantitative standards, calibration curves and R^2^ for the quantification of volatile compounds used in this study are provided in Table S4.

### Statistical analysis

Analysis of variance (ANOVA, least significant difference method at a significance level of *P* ≤ 0.05) was used to evaluate differences between the volatile and non-volatile compounds in the fermentation samples by the SPSS statistical package version 19.0 (SPSS Inc., USA).

## Results

### The cell growths, amino acid consumption, and main fermentation products

**Figure 1:**
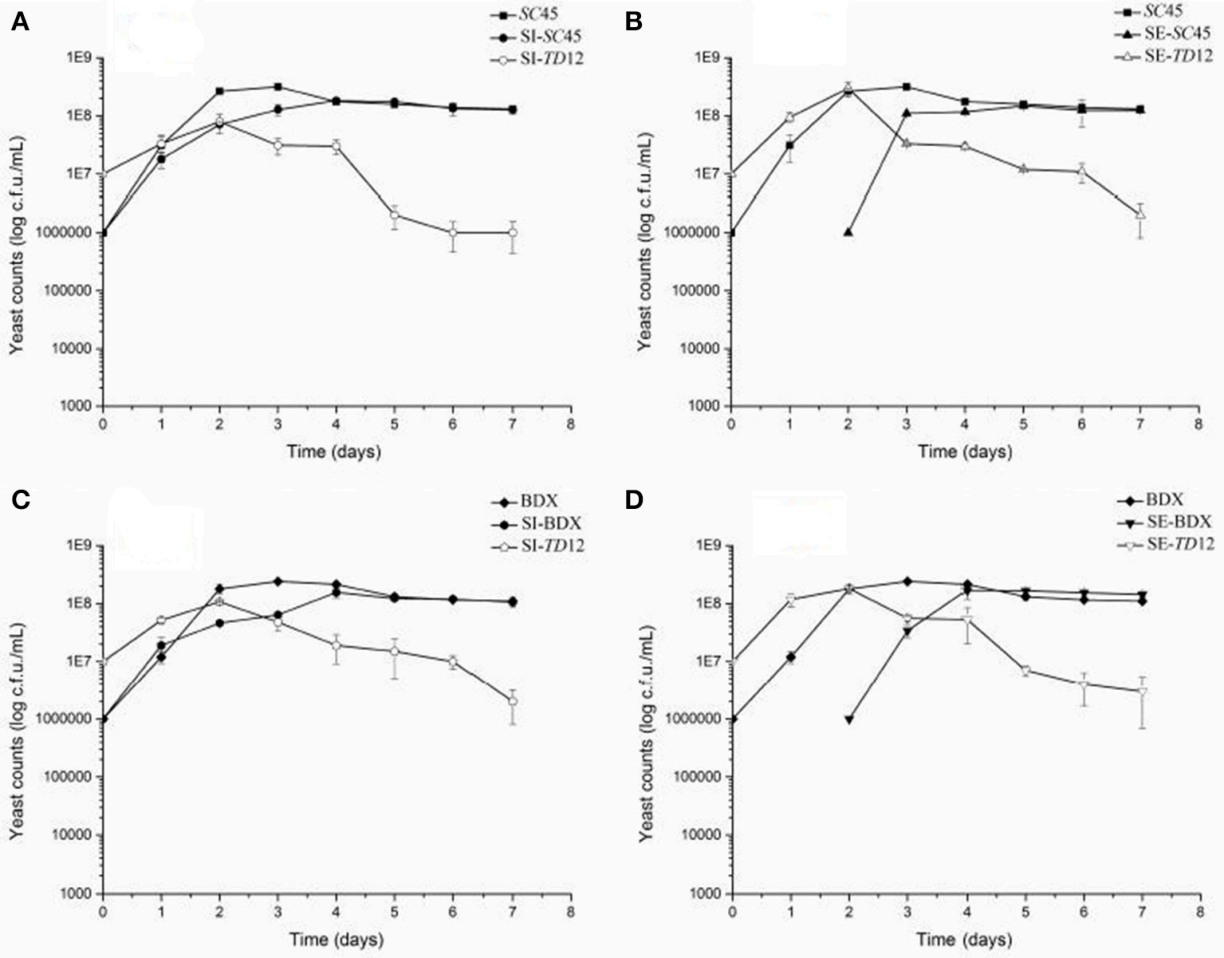
Yeast population dynamics in different fermentations **(A)** *SC*45 growth in pure fermentation (*SC45)* and the growths of *SC*45 and *TD*12 in simultaneous fermentation (SI-*SC*45 and SI-*TD*12, respectively); **(B)** *SC*45 growth in pure fermentation (*SC*45) and the growths of *SC*45 and *TD*12 in sequential fermentation (SE-*SC*45 and SE-*TD*12,respectively); **(C)** BDX growth in pure fermentation (BDX) and the growths of BDX and TD12 in simultaneous fermentation (SI-BDX and SI-*TD*12, respectively); **(D)** BDX growth in pure fermentation (BDX) and the growths of BDX and *TD*12 in sequential fermentation (SE-BDX and SE-*TD*12, respectively). Error bars represent the standard deviation.

The contribution of wine yeasts to the final aromatic characteristics of wine is largely depending on the persistence of strains and cell number during alcoholic fermentation. Figure 1 showed the growth dynamics of *S. cerevisiae* and *T. delbrueckii* in pure and mixed cultures. The amount of pure culture of *SC*45 increased rapidly during initial 2 days and reached the maximum number 3 × 10^8^ CFU/mL at the third days, and then decreased to 1 × 10^8^ CFU/mL and remain stable to the end of alcoholic fermentation. In the simultaneous fermentation, although the initial number of *SC*45 was lower than *TD*12, along the fermentation process, the cell number of *SC*45 exceeded *TD*12 at the third days (1.82 × 10^8^ CFU/mL). Similarly, in sequential fermentation, the cell number of *SC*45 exceed *TD*12 after 1 d fermentation (1.48 × 10^8^ CFU/mL) and remain steady till the end of alcoholic fermentation. It should be noticed that the highest populations of BDX and *SC*45 in sequential fermentation were comparable to those of simultaneous fermentation, suggesting that *TD*12 is not killer strain that secret toxin on *S. cerevisiae*, as reported by Velázquez et al. (2015). Lower population of *S. cerevisiae* in mixed fermentation compared to pure fermentation suggested that there is nutrition competition between both strains and negatively influenced individual strain growth. *T. delbrueckii* cells are less adapt to the grape must because in all cases, their number rapidly decreased after 2 d fermentation relative to *S. cerevisiae*, which were consistent with the data of previous literature (Loira et al., 2014).

The alcoholic fermentations ended after 7 d (pure fermentation) and 9 d (mixed fermentation). Residual glucose and fructose concentrations, and main fermentation products were showed in Table 1, including non-volatile acids, glycerol, acetic acid and ethanol. No glucose and 2.71–3.81 g/L fructose were left in wines, suggesting that all inoculations can successfully complete the alcoholic fermentation. Significantly decreased contents of acetic acid were observed in BDX/*TD*12 mixed fermentation (SE and SI) compared to pure fermentation, corresponding to the results that *T. delbrueckii* co-inoculation with *S. cerevisiae* efficiently reduced acetic acid content (Azzolini et al., 2015; Belda et al., 2015). However, this reduction was not occurred in *TD*12/*SC*45 mixed fermentation. Mixed fermentation also decreased ethanol contents compared to pure fermentation, including SI-*SC*45/*TD*12 (11.55 vs. 10.73%), SI-BDX/*TD*12 (11.57 vs. 11.01%) and SE-BDX/*TD*12 (11.57 vs. 11.10%). This was agreed with the results of Belda et al. (2015) that *T. delbrueckii* has the potential to reduce ethanol in winemaking. Sequential inoculation of BDX or *SC*45 can decrease succinic acid, which was inconsistent with the data that Puertas et al. (2016), who found that succinic acid concentration was 33% higher in 2011 Palomino wines in sequential fermentation of *Td*/*Sc* than pure *S. cerevisiae*. No clear differences were observed in other physiochemical parameters. After malolactic fermentation, acetic acid contents were slightly increased relative to alcoholic fermentation, except for SE-BDX/*TD*12, in which acetic acid was further decreased to 0.46 g/L. No significant differences were observed in other parameters among different wine samples.

**Table 1:** Analytical parameters (g/L) of the final wines after alcoholic fermentation.

**Compounds**	***SC*45**	**SI-*SC*45/*TD*12**	**SE-*SC*45/*TD*12**	**BDX**	**SI-BDX/*TD*12**	**SE-BDX/*TD*12**
Glucose	ND	ND	ND	ND	ND	ND
Fructose	3.15 ± 0.12^a^	2.81 ± 0.23^bc^	3.00 ± 0.15^b^	3.16 ± 0.07^a^	2.71 ± 0.18^c^	3.20 ± 0.10^a^
Glycerol	8.24 ± 0.18^ab^	8.18 ± 0.14^ab^	8.19 ± 0.25^bc^	8.87 ± 0.20^a^	8.57 ± 0.57^a^	8.67 ± 0.30^a^
Ethanol (%)	11.55 ± 0.14^a^	10.73 ± 0.41^b^	11.50 ± 0.42^a^	11.57 ± 0.09^a^	11.01 ± 0.02^b^	11.1 ± 0.11^b^
Citric acid	0.33 ± 0.04^a^	0.27 ± 0.04^a^	0.29 ± 0.01^a^	0.29 ± 0.04^a^	0.28 ± 0.04*a*	0.28 ± 0.01^a^
Tartaric acid	1.43 ± 0.19^b^	1.56 ± 0.16^a^	1.60 ± 0.10^a^	1.45 ± 0.08^b^	1.59 ± 0.04^a^	1.52 ± 0.01^bc^
Malic acid	6.83 ± 0.32^b^	6.97 ± 0.16^bc^	7.43 ± 0.99^a^	6.86 ± 0.72^b^	7.07 ± 0.49^bc^	7.46 ± 0.11^a^
Succinic acid	7.97 ± 0.29^ab^	7.16 ± 0.18^abc^	6.68 ± 0.05^c^	8.01 ± 0.80^a^	7.83 ± 0.22^ab^	6.96 ± 0.33^bc^
Lactic acid	0.89 ± 0.02^e^	1.07 ± 0.27^cd^	1.31 ± 0.44^b^	1.17 ± 0.16^c^	0.96 ± 0.10^d^	1.44 ± 0.14^a^
Acetic acid	0.61 ± 0.03^abc^	0.63 ± 0.02^ab^	0.66 ± 0.04^a^	0.63 ± 0.09^ab^	0.51 ± 0.01^c^	0.52 ± 0.01^bc^

*SC45, S. cerevisiae SC45 pure fermentation; SI-SC45/ TD12, Simultaneous inoculation of SC45 and TD12; SE-SC45/ TD12, Sequential inoculation of TD12 followed by SC45 after 2 days; BDX:S. cerevisiae BDX pure fermentation; SI-BDX/ TD12, Simultaneous inoculation of BDX and TD12; SE-BDX/ TD12, Sequential inoculation of TD12 followed by BDX after 2 days*.

Values are given as mean ± standard deviation of two biological replicates and three HPLC detection runs. Data with different superscript letters (a, b, c, d, e, f) within each column are different according to Duncan tests (0.05%). ND, undetected

Amino acids are important nitrogen for wine yeast growth and fermentation activity. More important, their composition and amount significantly affect the formation of many volatile compounds that contribute to wine flavor. To evaluate the effects of different multi-cultures on the discrepancies of amino acid metabolism, the consumption profiles of amino acid during alcoholic fermentation were calculated by the ratio of consumed content to the initial content (Table 2). In addition, the changed ratios of amino acid after malolactic fermentation *vs* alcoholic fermentation were determined by comparing the individual residual content (Table S1). Except for Pro, most amino acids were totally consumed during alcoholic fermentation, but were largely dependent on the type of amino acid and inoculation method. The consumption of Asn, β-Ala, Tyr, Met, Leu, Trp, Phe, Orn, and Lyr, especially Asn, Tyr, Met, Trp, Phe, and Orn, were pronounced higher in mixed fermentation than pure fermentation. After malolactic fermentation, most amino acids in wines were increased compared to the wines after alcoholic fermentation, which might be due to the collapse of yeast cell and the excretion of amino acid into wines (Hernandezorte et al., 2006). Conversely, the contents of Asn, Arg and Ile were further decreased by the metabolism of lactic acid bacterial. Lowest total YAN content were found in SI fermentation compared to other inoculations, but there were no clearly regular profiles.

**Table 2:** The consumption ratio of nitrogenous compounds after alcoholic fermentation(%).

**Nitrogenous compounds**	***SC*45**	**SI-*SC*45/*TD*12**	**SE-*SC*45/*TD*12**	**BDX**	**SI-BDX/*TD*12**	**SE-BDX/*TD*12**
Asp	91.54 ± 3.18	89.47 ± 2.94	93.40 ± 0.48	94.65 ± 2.17	93.01 ± 4.18	93.22 ± 2.83
Glu	69.52 ± 5.49	66.25 ± 2.57	68.01 ± 1.51	67.93 ± 2.86	69.62 ± 2.97	69.83 ± 0.34
Ser	77.77 ± 11.90	74.90 ± 1.83	76.64 ± 0.62	75.67 ± 3.05	74.65 ± 2.03	76.04 ± 1.44
Asn	48.3 ± 32.83	73.6 ± 2.09	75.27 ± 1.10	68.95 ± 4.39	74.18 ± 1.67	78.72 ± 1.20
Gln	69.19 ± 19.45	62.23 ± 1.64	65.20 ± 1.95	62.44 ± 1.96	62.09 ± 0.40	63.70 ± 1.27
His	87.36 ± 2.20	88.56 ± 2.47	91.49 ± 0.51	91.73 ± 0.69	92.90 ± 1.03	93.21 ± 0.47
Gly	79.89 ± 2.09	82.03 ± 1.19	82.62 ± 0.57	81.54 ± 0.29	83.08 ± 0.93	83.30 ± 0.53
Thr	94.48 ± 2.52	97.13 ± 0.48	96.20 ± 0.10	97.09 ± 0.37	97.32 ± 0.73	97.76 ± 0.18
β-Ala	60.61 ± 12.55	69.7 ± 1.66	66.61 ± 3.32	64.16 ± 3.97	71.22 ± 1.71	69.70 ± 1.15
Arg	96.33 ± 2.23	95.00 ± 0.66	95.77 ± 0.87	96.07 ± 0.38	96.74 ± 0.71	96.90 ± 0.44
Ala	97.36 ± 0.72	94.77 ± 0.69	96.86 ± 1.97	97.71 ± 0.15	97.05 ± 0.52	96.82 ± 0.64
GABA	89.16 ± 1.88	84.04 ± 2.22	87.58 ± 0.93	90.84 ± 1.07	90.37 ± 1.36	90.47 ± 1.27
Pro	−459.40 ± 30.80	−394.30 ± 5.90	−402.30 ± 4.40	−439.50 ± 13.20	−398.60 ± 14.10	−401.64 ± 10.10
NH4^+^	93.62 ± 1.26	95.06 ± 0.76	96.91 ± 1.74	94.78 ± 0.47	96.00 ± 0.14	96.09 ± 0.30
Tyr	23.53 ± 22.45	52.35 ± 7.45	10.59 ± 10.39	41.27 ± 14.80	50.10 ± 0.98	53.33 ± 1.08
Val	88.89 ± 2.39	90.00 ± 0.78	89.98 ± 0.40	91.32 ± 1.71	90.19 ± 0.38	90.53 ± 0.27
Met	24.91 ± 26.46	52.41 ± 0.69	52.41 ± 10.82	1.89 ± 60.31	38.66 ± 6.01	32.3 ± 10.65
Cys	13.60 ± 45.10	32.97 ± 45.60	88.36 ± 3.55	77.08 ± 12.25	71.2 ± 22.30	80.88 ± 12.50
Ile	83.26 ± 5.60	87.53 ± 2.85	86.41 ± 2.07	86.38 ± 2.94	86.20 ± 2.48	88.02 ± 1.73
Leu	78.85 ± 2.64	83.03 ± 1.77	83.03 ± 1.21	82.16 ± 1.13	85.56 ± 1.43	87.03 ± 1.77
Trp	49.26 ± 5.65	62.36 ± 4.88	63.84 ± 1.09	57.35 ± 8.67	63.01 ± 3.08	61.14 ± 9.12
Phe	69.94 ± 9.01	82.91 ± 5.73	79.47 ± 1.48	77.78 ± 5.58	80.49 ± 3.08	81.22 ± 1.81
Orn	46.36 ± 13.38	60.6 ± 7.82	73.45 ± 2.36	60.49 ± 8.57	71.52 ± 12.85	82.87 ± 5.35
Lyr	65.06 ± 19.51	77.01 ± 9.50	65.73 ± 2.37	71.16 ± 4.92	87.19 ± 10.18	86.26 ± 6.53
YAN	89.21 ± 2.03	89.30 ± 0.82	90.68 ± 0.68	90.26 ± 0.51	91.12 ± 0.51	91.57 ± 0.35

*SC45, S. cerevisiae SC45 pure fermentation; SI-SC45/TD12, Simultaneous inoculation of SC45 and TD12; SE-SC45/TD12, Sequential inoculation of TD12 followed by SC45 after 2 days; BDX:S. cerevisiae BDX pure fermentation; SI-BDX/ TD12, Simultaneous inoculation of BDX and TD12; SE-BDX/TD12, Sequential inoculation of TD12 followed by BDX after 2 days*.

*The data were calculated by the ratio of consumed content to the initial content in the must*.

*All data are shown as mean values ± standard deviations*.

### Volatile aroma compounds after alcoholic fermentation and malolactic fermentation

The aromatic profiles of the wine produced by different inoculated strains and methods were determined. Generally, pure fermentation of *SC*45 produced higher levels of higher alcohols, fatty acids and fatty acid ethyl esters compared to BDX. Totally, forty-four aroma compounds in final wines were identified after alcoholic and malolactic fermentation, including fifteen higher alcohols, three fatty acids, sixteen esters (four acetate esters, eight fatty acid ethyl esters and four other esters), three aldehydes, four terpenes and three volatile phenols (Table 3, Table S2). The compound that odor activity value (OAV) over one have high contribution to wine aroma (Guth, 1997). Thus, the aroma compounds in Table 3 that OAV exceeding one were highlighted and underlined, and those compounds that OAV exceeding 0.1 but <1 were underlined. To evaluate the effect of malolactic fermentation on aroma compounds development, the ratios of aroma compound contents (OAV>0.1) after malolactic fermentation vs. alcoholic fermentation were calculated and showed in Table 4.

**Table 3:** Volatile composition(μg/L) of the final wines after alcoholic fermentation.

**Aroma compounds**	***SC*45**	**SI-*SC*45/*TD*12**	**SE-*SC*45/*TD*12**	**BDX**	**SI-BDX/*TD*12**	**SE-BDX/*TD*12**
1-Hexanol	**5241.78 ± 369.18^abc^**	**5192.89 ± 388.75^abc^**	**5562.65 ± 119.41^ab^**	**4922.56 ± 416.52^c^**	**5083.62 ± 466.31^bc^**	**5721.11 ± 164.72^a^**
(E)-3-Hexen-1-ol	66.52 ± 29.88^cd^	79.09 ± 24.71^b^	91.35 ± 3.29^a^	68.21 ± 2.52^c^	56.75 ± 18.33^d^	76.74 ± 34.71^b^
(Z)-3-Hexen-1-ol	127.27 ± 1.21^*cd*^	150.53 ± 10.41^b^	191.24 ± 5.35^a^	120.43 ± 3.07^d^	126.41 ± 9.15^*cd*^	132.75 ± 4.69^c^
**Total C**_6_ **alcohols**	5435.58 ± 400.26^b^	5422.51 ± 423.87^b^	5845.25 ± 128.04^a^	5111.18 ± 422.14^d^	5266.72 ± 493.78^c^	5930.55 ± 204.12^a^
3-Methyl-1-butanol	**159642.8 ± 8266.1^c^**	**170810.4 ± 16962.9^b^**	**216990.5 ± 13317.5^a^**	**149572.5 ± 6036.6^d^**	**176139.1 ± 23431.5^b^**	**228511.1 ± 15335.1^a^**
3-Methyl-1-pentanol	227.75 ± 9.43^ab^	214.25 ± 21.54^b^	227.91 ± 30.76^ab^	225.35 ± 12.85^ab^	249.63 ± 20.06^a^	218.05 ± 16.28^ab^
4-Methyl-1-pentanol	5.78 ± 0.41^bc^	5.02 ± 0.46^d^	5.82 ± 0.61^bc^	5.33 ± 0.32^cd^	6.64 ± 0.62^a^	6.31 ± 0.52^ab^
2-Octanol	1.92 ± 0.19^a^	1.52 ± 0.16^b^	1.85 ± 0.12^ab^	1.76 ± 0.32^ab^	1.56 ± 0.23^b^	1.77 ± 0.15^ab^
1-Octen-3-ol	11.15 ± 0.64^a^	10.55 ± 1.42^a^	11.74 ± 0.52^a^	10.94 ± 0.76^a^	9.21 ± 1.08^b^	10.99 ± 0.41^a^
2-Ethyl-1-hexanol	3.15 ± 0.45^b^	3.54 ± 0.72^b^	3.89 ± 0.67^b^	3.12 ± 0.24^b^	5.24 ± 0.63^a^	4.17 ± 1.03^b^
2-Nonanol	1.39 ± 0.19^b^	1.41 ± 0.29^b^	1.85 ± 0.08^b^	1.49 ± 0.34^b^	1.51 ± 0.39^b^	2.55 ± 0.28^a^
1-Octanol	253.18 ± 25.09^c^	266.78 ± 96.06^c^	351.73 ± 16.36^a^	253.85 ± 13.05^c^	335.66 ± 99.57^b^	355.39 ± 82.06^a^
(6Z)-Nonen-1-ol	7.88 ± 1.28^ab^	6.81 ± 0.53^b^	7.44 ± 0.19^ab^	8.16 ± 0.66^a^	7.06 ± 0.38^b^	7.81 ± 0.14^ab^
1-Decanol	5.68 ± 0.39^b^	6.23 ± 1.36^b^	12.86 ± 0.52^a^	5.67 ± 0.46^b^	7.59 ± 1.68^b^	14.13 ± 2.17^a^
Benzyl alcohol	385.02 ± 51.38^b^	297.87 ± 29.19^b^	336.89 ± 28.54^a^	350.36 ± 21.78^b^	258.21 ± 32.82^b^	295.14 ± 10.95^b^
Phenylethyl alcohol	**165160.3 ± 30126.9^bcd^**	**189373.4 ± 16616.3^b^**	**243967.3 ± 29508.1^a^**	**136500.7 ± 12257.6^d^**	**143542.1 ± 25766.7^cd^**	**177118.4 ± 9102.7^bc^**
**Total of higher alcohols**	331141.6 ± 38227.7^bcd^	366420.3 ± 33509.6^b^	469248.0 ± 42758.5^a^	292050.4 ± 18151.6^d^	325830.1 ± 48745.5^cd^	412476.3 ± 57084.9^ab^
Hexanoic acid	**1769.04 ± 232.52^a^**	**1309.73 ± 18.79^b^**	**1392.02 ± 56.71^b^**	**1320.72 ± 55.87^b^**	**1469.21 ± 151.54^b^**	**1291.12 ± 52.92^b^**
Octanoic acid	**1864.44 ± 340.71^ab^**	**1564.43 ± 122.98^c^**	**1932.77 ± 130.82^a^**	**1658.99 ± 133.48^abc^**	**1608.97 ± 166.03^bc^**	**1442.38 ± 100.81^c^**
n-Decanoic acid	208.61 ± 9.68^bc^	212.52 ± 2.81^b^	236.63 ± 9.08^a^	202.24 ± 5.93^c^	207.88 ± 4.57^bc^	213.61 ± 1.89^b^
**Total of fatty acids**	3842.08 ± 498.33^a^	3086.68 ± 143.35^c^	3561.44 ± 194.15^ab^	3181.91 ± 180.45^bc^	3286.15 ± 207.84^bc^	2947.12 ± 155.49^c^
Isoamyl acetate	**1603.07 ± 224.18^bc^**	**1513.86 ± 226.75^bc^**	**2189.93 ± 56.07^a^**	**1426.39 ± 261.89^c^**	**1821.27 ± 169.21^b^**	**2173.32 ± 288.33^a^**
Hexyl acetate	12.63 ± 1.82^a^	8.11 ± 1.88^b^	12.26 ± 0.45^a^	12.21 ± 3.77^a^	9.49 ± 2.28^ab^	8.77 ± 2.83^ab^
Phenethyl acetate	73.29 ± 4.34^b^	70.22 ± 11.49^b^	88.31 ± 8.91^a^	69.65 ± 7.76^b^	78.63 ± 8.71^ab^	78.41 ± 10.12^ab^
Ethyl acetate	**56607.92 ± 5421.21^b^**	**51641.06 ± 5544.05^b^**	**55320.26 ± 2118.52^b^**	**69189.61 ± 1648.02^a^**	**58893.92 ± 3760.55^b^**	**74282.24 ± 6428.22^a^**
**Total of acetate esters**	58296.91 ± 5643.53^bc^	53233.23 ± 5758.26^c^	57610.77 ± 2125.05^bc^	70697.85 ± 1452.97^a^	60803.31 ± 3895.52^b^	76542.73 ± 6177.28^a^
Ethyl dodecanate	38.09 ± 1.47^b^	36.4 ± 1.02^b^	42.64 ± 1.08^a^	37.85 ± 1.41^b^	38.04 ± 2.03^b^	43.08 ± 1.07^a^
Ethyl butanoate	**502.45 ± 36.29^bc^**	**473.06 ± 46.02^c^**	**550.94 ± 18.97^ab^**	**494.43 ± 13.15^bc^**	**506.36 ± 29.44^bc^**	**568.56 ± 51.25^a^**
Ethyl hexanoate	**355.01 ± 33.96^a^**	**332.52 ± 28.07^a^**	**374.87 ± 13.89^a^**	**347.15 ± 34.41^a^**	**356.78 ± 22.75^a^**	**359.61 ± 27.99^a^**
Ethyl heptanoate	0.21 ± 0.02^b^	0.19 ± 0.04^b^	0.34 ± 0.03^a^	0.21 ± 0.04^b^	0.19 ± 0.04^b^	0.29 ± 0.04^a^
Ethyl lactate	3562.54 ± 196.03^b^	4070.65 ± 348.52^a^	3585.11 ± 342.37^b^	2809.51 ± 100.25^c^	3142.25 ± 135.16^c^	2924.45 ± 171.19^c^
Ethyl octanoate	33.58 ± 2.29^c^	36.36 ± 2.01^c^	48.55 ± 1.73^a^	35.26 ± 0.57^c^	41.29 ± 2.61^b^	50.75 ± 2.35^a^
Ethyl nonanoate	0.82 ± 0.01^d^	0.91 ± 0.04^c^	1.08 ± 0.03^c^	0.91 ± 0.01^b^	1.03 ± 0.09^ab^	1.14 ± 0.04^a^
Ethyl decanoate	**860.94 ± 7.12^e^**	**879.11 ± 7.07^d^**	**963.79 ± 10.53^b^**	**865.14 ± 3.11^de^**	**906.45 ± 15.41^c^**	**995.17 ± 8.65^a^**
**Total of ethyl esters**	5355.92 ± 264.04^bc^	5832.16 ± 425.82^a^	5573.51 ± 339.71^ab^	4591.41 ± 50.93^d^	4995.19 ± 193.82^cd^	4948.97 ± 189.94^cd^
Isoamyl octanoate	3.32 ± 0.18^d^	3.79 ± 0.14^c^	4.92 ± 0.22^b^	3.65 ± 0.09^cd^	4.79 ± 0.38^b^	6.04 ± 0.39^a^
Diethyl succinate	46.58 ± 6.51^bc^	52.87 ± 3.74^b^	65.77 ± 1.91^a^	39.95 ± 3.56^c^	44.73 ± 7.98^c^	60.95 ± 2.37^a^
Methyl octanoate	0.69 ± 0.08^c^	0.69 ± 0.06^c^	1.11 ± 0.04^a^	0.68 ± 0.02^c^	0.76 ± 0.07^c^	0.98 ± 0.05^b^
Isopentyl hexanoate	2.94 ± 0.11^d^	3.12 ± 0.08^d^	3.66 ± 0.07^b^	2.96 ± 0.04^d^	3.46 ± 0.13^c^	3.95 ± 0.23^a^
**Total of other esters**	54.24 ± 6.77^bc^	61.19 ± 4.03^b^	76.09 ± 1.91^a^	47.86 ± 3.47^c^	54.34 ± 8.42^bc^	72.51 ± 2.03^a^
Decanal	2.48 ± 0.65^a^	1.49 ± 0.49^b^	1.72 ± 0.44^ab^	2.19 ± 0.62^ab^	1.76 ± 0.76^ab^	1.77 ± 0.51^ab^
Benzaldehyde	23.02 ± 1.23^ab^	23.12 ± 2.15^ab^	23.71 ± 1.93^a^	22.37 ± 0.73^ab^	21.32 ± 1.02^b^	20.88 ± 0.86^b^
Phenylacetaldehyde	**814 ± 158.22^c^**	**1054.1 ± 73.19^b^**	**1358.37 ± 166.22^a^**	**725.73 ± 71.05^c^**	**1042.12 ± 73.46^b^**	**1052.72 ± 55.58^b^**
**Total of aldehydes**	839.49 ± 158.98^c^	1078.71 ± 75.34^b^	1383.85 ± 167.84^a^	750.28 ± 71.76^c^	1065.24 ± 73.04^b^	1075.36 ± 55.19^b^
Linalool	1.13 ± 0.12^b^	1.13 ± 0.21^b^	1.41 ± 0.06^a^	1.07 ± 0.06^b^	1.13 ± 0.04^b^	1.19 ± 0.06^b^
Citronellol	10.35 ± 0.94^d^	11.68 ± 0.76^bc^	13.42 ± 1.09^a^	10.18 ± 0.74^d^	10.99 ± 0.71^*cd*^	12.65 ± 0.31^ab^
Geraniol	**20.72 ± 0.83^a^**	**20.17 ± 0.16^ab^**	**20.15 ± 0.09^ab^**	**20.62 ± 0.12^ab^**	**20.09 ± 0.11^b^**	**20.41 ± 0.31^ab^**
Farnesol	22.44 ± 0.49^ab^	21.75 ± 0.45^b^	22.01 ± 0.48^ab^	22.65 ± 0.43^a^	22.13 ± 0.56^ab^	21.97 ± 0.45^ab^
**Total of terpenes**	94.02 ± 8.34^b^	87.65 ± 6.49^b^	109.03 ± 2.49^a^	93.81 ± 5.14^b^	95.61 ± 12.44^b^	98.64 ± 10.05^a^
Phenol	78.01 ± 10.64^ab^	61.18 ± 6.98^c^	82.73 ± 4.61^a^	71.27 ± 6.45^abc^	66.08 ± 12.23^bc^	65.65 ± 5.86^bc^
4-Ethyl guaiacol	**36.71 ± 5.94^ab^**	**29.64 ± 4.62^b^**	**39.88 ± 2.52^a^**	**36.26 ± 1.58^ab^**	**32.77 ± 8.14^ab^**	**36.81 ± 4.95^ab^**
4-Ethyl-phenol	61.25 ± 15.99^a^	43.15 ± 3.34^b^	68.02 ± 11.94^a^	54.34 ± 3.49^ab^	51.45 ± 13.76^ab^	54.97 ± 2.44^ab^
**Total of volatile phenols**	175.92 ± 31.71^ab^	133.97 ± 14.74^c^	190.64 ± 16.66^a^	161.86 ± 10.36^abc^	150.34 ± 33.98^bc^	157.43 ± 13.08^abc^

*SC45, S. cerevisiae SC45 pure fermentation; SI-SC45/TD12, Simultaneous inoculation of SC45 and TD12; SE-SC45/TD12, Sequential inoculation of TD12 followed by SC45 after 2 days; BDX:S. cerevisiae BDX pure fermentation; SI-BDX/TD12, Simultaneous inoculation of BDX and TD12; SE-BDX/TD12, Sequential inoculation of TD12 followed by BDX after 2 days*.

*The aroma compounds (OVA>1) were highlighted and underlined, other volatile compounds (OVA>0.1) were underlined. Values are given as mean ± standard deviation of two biological replicates and three detection runs. Data with different superscript letters (a, b, c, d, e, f) within each column are different according to Duncan tests (0.05%)*.

**Table 4:** The ratio of aroma compounds (OVA > 0.1) after malolactic fermentation *vs*. after alcoholic fermentation.

**Aroma compounds**	***SC*45**	**SI-*SC*45/*TD*12**	**SE-*SC*45/*TD*12**	**BDX**	**SI-BDX/*TD*12**	**SE-BDX/*TD*12**
**ALCOHOLS**						
3-Methyl-1-butanol	1.01	1.11	0.97	1.03	1.08	1.01
3-Methyl-1-pentanol	1.01	1.09	0.93	1.02	0.95	1.00
1-Hexanol	1.02	0.98	0.94	1.04	1.00	0.98
(Z)-3-Hexen-1-ol	1.10	0.98	1.02	1.18	1.12	1.18
1-Octen-3-ol	1.23	1.11	1.03	1.32	1.33	1.12
1-Octanol	0.91	1.01	0.86	0.97	0.77	0.96
Phenylethyl alcohol	0.75	1.15	0.90	0.77	1.44	1.18
**FATTY ACIDS**						
Hexanoic acid	0.94	1.40	1.06	1.21	1.10	1.26
Octanoic acid	1.13	1.89	1.21	1.22	1.52	1.64
n-Decanoic acid	1.05	1.13	1.03	1.01	1.12	1.18
**ESTERS**						
Isoamyl acetate	0.69	0.77	0.58	0.75	0.64	0.48
Ethyl acetate	1.11	1.09	0.84	0.98	0.93	0.87
Ethyl butanoate	0.87	1.01	0.78	0.98	0.82	0.82
Ethyl hexanoate	1.00	1.10	1.04	0.99	0.99	0.94
Ethyl decanoate	1.02	1.04	1.02	1.00	0.99	1.01
**TERPENES**						
Citronellol	1.05	1.11	0.97	1.13	1.17	1.10
Geraniol	0.99	1.04	1.01	0.99	1.02	1.01
**ALDEHYDES**						
Decanal	1.04	1.74	1.33	1.13	2.19	1.19
Phenylacetaldehyde	0.77	0.85	0.66	0.84	0.80	0.83
**PHENOLS**						
4-Ethyl guaiacol	0.87	1.24	1.00	0.99	1.03	1.18
4-Ethyl-phenol	0.76	1.26	0.89	1.09	0.95	1.07
Total	1.07	1.25	1.05	1.09	1.26	1.13

*SC45, S. cerevisiae SC45 pure fermentation; SI-SC45/TD12, Simultaneous inoculation of SC45 and TD12; SE-SC45/TD12, Sequential inoculation of TD12 followed by SC45 after 2 days; BDX:S. cerevisiae BDX pure fermentation; SI-BDX/TD12, Simultaneous inoculation of BDX and TD12; SE-BDX/TD12, Sequential inoculation of TD12 followed by BDX after 2 days*.

*The values were calculated from the ratio of aroma compound contents (OAV>0.1) after malolactic fermentation vs alcoholic fermentation*.

### C_6_ Alcohol and higher alcohols

C_6_ alcohols usually have the character of “vegetal” and “herbaceous” note, giving negative effect on wine aroma quality (Ferreira et al., 2000). Three C_6_ alcohols (1-hexanol, Z-3-hexen-1-o, E-3-hexen-1-ol) were identified. Mixed fermentation caused significant increment of total C_6_ alcohols, 1-hexanol and Z-3-hexen-1-ol contents, especially sequential fermentation, and this characteristic retained in malolactic fermentation as well. Although *SC*45 generated more C_6_ alcohols than BDX in pure fermentation, the highest amount of 1-hexanol and total C_6_ alcohols were produced in SE-BDX/*TD*12.

The higher alcohols were the largest group of aroma compounds in this study. The concentration of 300–400 mg/L is acceptable, and the optimal level (below 300 mg/L) give a pleasant character (Rapp and Versini, 1995). In this work, the total concentrations of this group compound are ranged in 292.05 mg/L (BDX) to 469.25 mg/L (SE-*SC*45/*TD*12) after alcoholic fermentation and 292.68 mg/L (*SC*45) to 445.86 mg/L (SE-BDX/*TD*12) after malolactic fermentation. 3-Methyl-1-butanol, phenylethyl alcohol (contributing to “flowery” and “sweet” notes for wine) and 3-methyl-1-pentanol were detected above their threshold. Monoculture *SC*45 produced higher concentration of high alcohol than BDX. This trait further maintained in mixed fermentation, and SE-*SC*45/*TD*12 generated the highest amount of total, 3-methyl-1-pentanol and phenylethyl alcohol, followed by SE-BDX/*TD*12, SI-*SC*45/*TD*12 and SE-BDX/*TD*12. The malolactic fermentation can increase or decrease higher alcohol levels, depending on inoculated *S. cerevisiae* strains and inoculation method. It should be noticed that the concentration of phenylethyl alcohol in SI-BDX/*TD*12 wine was increased by 1.44 folds after malolactic fermentation compared to the value of alcoholic fermentation.

### Fatty acids

Fatty acids imparts the wine with fruity, cheese, fatty and rancid notes (Schreier and Jennings, 1979). Volatile fatty acids can improve the complexity of wine at sub-sensory threshold levels, but cause negative effect on wine aroma when above their thresholds (Swiegers and Pretorius, 2005). Hexanoic acid, octanoic acid and n-decanoic acid were detected in this study, and the former two compounds exceeded individual threshold. Lower production of fatty acid is particularly trait in single culture of *T. delbrueckii* and mixed fermentation, and the co-cultures of *T. delbrueckii* and *Saccharomyces* yeasts can modulate the production of fatty acids (Azzolini et al., 2012, 2015). Similar results were observed in this study, but inoculated *S. cerevisiae* strain largely influenced their formation. 26.0, 16.1, and 19.7% decreased contents of hexanoic acid, octanoic acid and total contents were observed in SI-*SC*45/*TD*12 than pure *SC*45 fermentation, respectively, but this decrement was not observed in mixed fermentation of BDX/*TD*12. Fatty acid contents were significantly promoted by malolactic fermentation; especially in mixed fermentation wines, for example, octanoic acid in SI-*SC*45/*TD*12 and SE-*SC*45/*TD*12 wines were 1.89 and 1.64 folds higher than the values of alcoholic fermentation, respectively.

### Esters

Esters, including acetate esters and ethyl esters, are important aroma compounds and positively contribute to the desired fruit aroma characters of wine. In this study, sixteen esters were identified and five esters reached their threshold, including isoamyl acetate, ethyl acetate, ethyl butanoate, ethyl hexanoate, and ethyl decanoate. Compared to *SC*45, BDX is better strain that produce acetate esters, which was evidenced in sequential fermentation because significant enhancement of isoamyl acetate (banana note) was achieved in SE-BDX/*TD*12. However, this promotion was not occurred in *SC*45/*TD*12. Similarly, *SC*45 produced higher amount of ethyl esters than BDX, especially ethyl lactate (generating descriptors of coffee and strawberry), and SI-*SC*45/*TD*12 generated the highest contents of ethyl esters. The promotion of multi-culture of *T. delbrueckii* and *S. cerevisiae* on ethyl esters was reported by Sun et al. (2014) as well. The malolactic fermentation decreased most esters contents relative to alcoholic fermentation, except for ethyl hexanoate and ethyl decanoate. It should be noticed that this reduction was pronounced occurred in the wines of sequential inoculation, for example, isoamyl acetate contents in SE-*SC*45/*TD*12 and SE-BDX/*TD*12 were 0.42 and 0.52 folds lower than those of alcoholic fermentation, respectively, while the corresponding values were 0.31 and 0.25 folds in pure fermentation, respectively.

### Aldehydes

Aldehydes with low sensory threshold and apple-like odors are important to wine aroma. Three aldehydes were identified in this study, and only phenylacetaldehyde (generating floral and honey smell in wine) reached OAV over one. Phenylacetaldehyde was promoted by mixed fermentation compared to pure *S. cerevisiae*, especially sequential fermentation. The highest value (1358.37 vs. 814.25 μg/L) was obtained in SE-*SC*45/*TD*12 compared to pure fermentation, which was corresponding to the data that *SC*45 produced more phenylacetaldehyde than BDX (814.25 vs. 725.73 μg/L). The level of this compound was decreased after malolactic fermentation, which was more pronounced in SE-*SC*45/*TD*12.

### Terpenes and volatile phenols

Terpenes provide floral notes to wine and have low odor thresholds. Most terpenes are rich in aromatic grape varieties. Although Cabernet Sauvignon does not belong to aromatic varieties and has a low content of terpenes, four terpenes were still detected in final wines including linalool, citronellol, geraniol and farnesol, in which, only geraniol exceeded its threshold. *SC*45 and BDX showed comparable promotion on terpenes formation when combined with *TD*12 inoculation, and the higher total amounts were obtained in SE-*SC*45/*TD*12. Generally, the beneficial effects of mixed fermentation on terpenes formation were amplified by malolactic fermentation (Table S2).

Decreased volatile phenols content is another characteristic of multi-culture of *T. delbrueckii* and *S. cerevisiae* (Renault et al., 2009; Azzolini et al., 2015). In this study, the contents of 4-ethyl guaiacol and 4-ethyl-phenol were significantly lower in SI-*SC*45/*TD*12 than those of pure fermentation, but this reduction was not observed in other mixed fermentations. The contents of volatile phenols showed increment after malolactic fermentation, especially in SI-*SC*45/*TD*12 wine.

**Figure 2:**
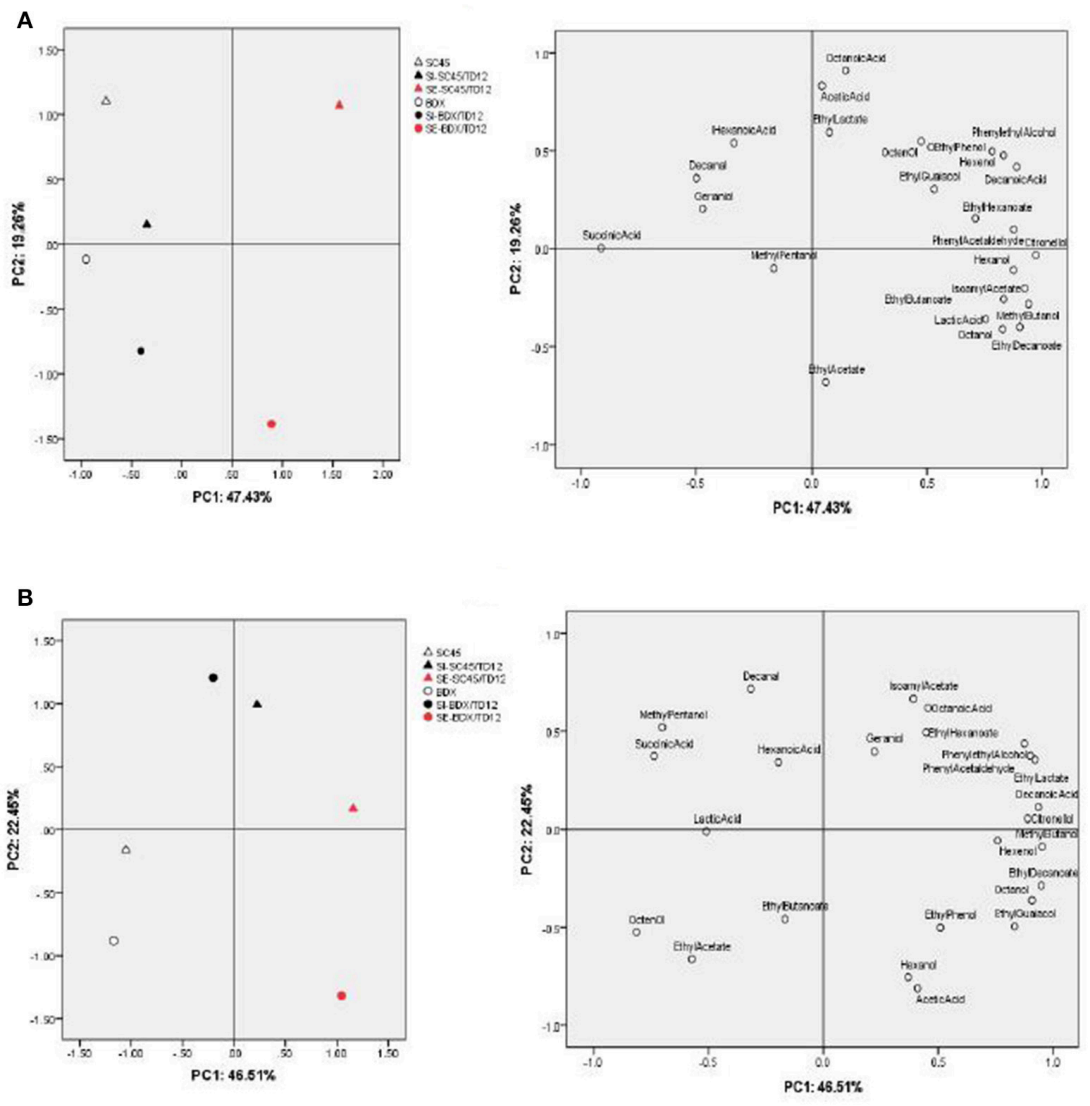
Principal component analysis (PCA) biplots of wines resulting from 22 aromatic compounds (OAV above 0.1) and 3 organic acid (succinic acid, lactic acid, acetic acid) by different fermentation methods after alcoholic fermentation **(A)** and malolactic fermentation **(B)**, respectively.

The above data indicated that co-fermentation of *T. delbrueckii* with different *S. cerevisiae* strain (*SC*45 and BDX) could produce diversified volatile profile of wine, which was not only dependent on *S. cerevisiae* strains paired inoculation, but also on inoculation methods (SI and SE). To highlight the differences between wines fermented by different *S. cerevisiae* strains and inoculation methods, and to identify the volatile compounds that discriminate these treatments, Principal Component Analysis (PCA) was applied using main physical chemical and aromatic compound that OAV exceed 0.1 after alcoholic fermentation and malolactic fermentation, respectively (Table 3 and Table S2). The PCA in Figure 2A indicated that the wines of alcoholic fermentation can be clearly separated by the first two components (47.43% for PC1 and 19.26% for PC2). The wines obtained from *SC*45 monoculture were clearly separated from BDX, suggesting that SC45 and BDX have dissimilarity among their formation of aromatic compounds. This characteristic led to the diversity of simultaneous and sequential wines. The wine of SI-*SC*45/*TD*12 was located in the positive part of PC2, and the wine of SI-BDX/*TD*12 located in the negative part of PC2. Monoculture and simultaneous wines are located together suggested that both inoculation have similar aromatic profiles. The significant differences were found in sequential wines, which were far from each other and from other inoculations, SE-*SC*45/*TD*12 wine were characteristic with phenylethyl alcohol, decanoic acid, Z-3-hexen-1-ol, while ethyl acetate were specific for the wine of SE-BDX/*TD*12. These results confirmed the previous data that sequential fermentation of *T. delbrueckii* and *S. cerevisiae* is a better inoculation method because it can produce more desired aroma compounds compared to simultaneous fermentation (Taillandier et al., 2014). The PCA in Figure 2B indicated that after malolactic fermentation, the differences of aromatic profiles of all wines were reduced, especially in monoculture wine. However, compared to simultaneous wines, sequential wines still had higher diversified aroma profiles. The main responsible components for the separation of SE-*SC*45/*TD*12 and SE-BDX/*TD*12 wines were succinic acid, 3-hexen-1-ol, isoamyl acetate, ethyl hexanoate, and ethyl acetate. In comparison, the main principal components responsible for discriminating SI-*SC*45/*TD*12 and SI-BDX/*TD*12 were acetic acid, hexanoic acid, octanoic acid, ethyl butanoate, and decanal.

### Influence of different inoculation methods on odor profile of final wine

**Figure 3:**
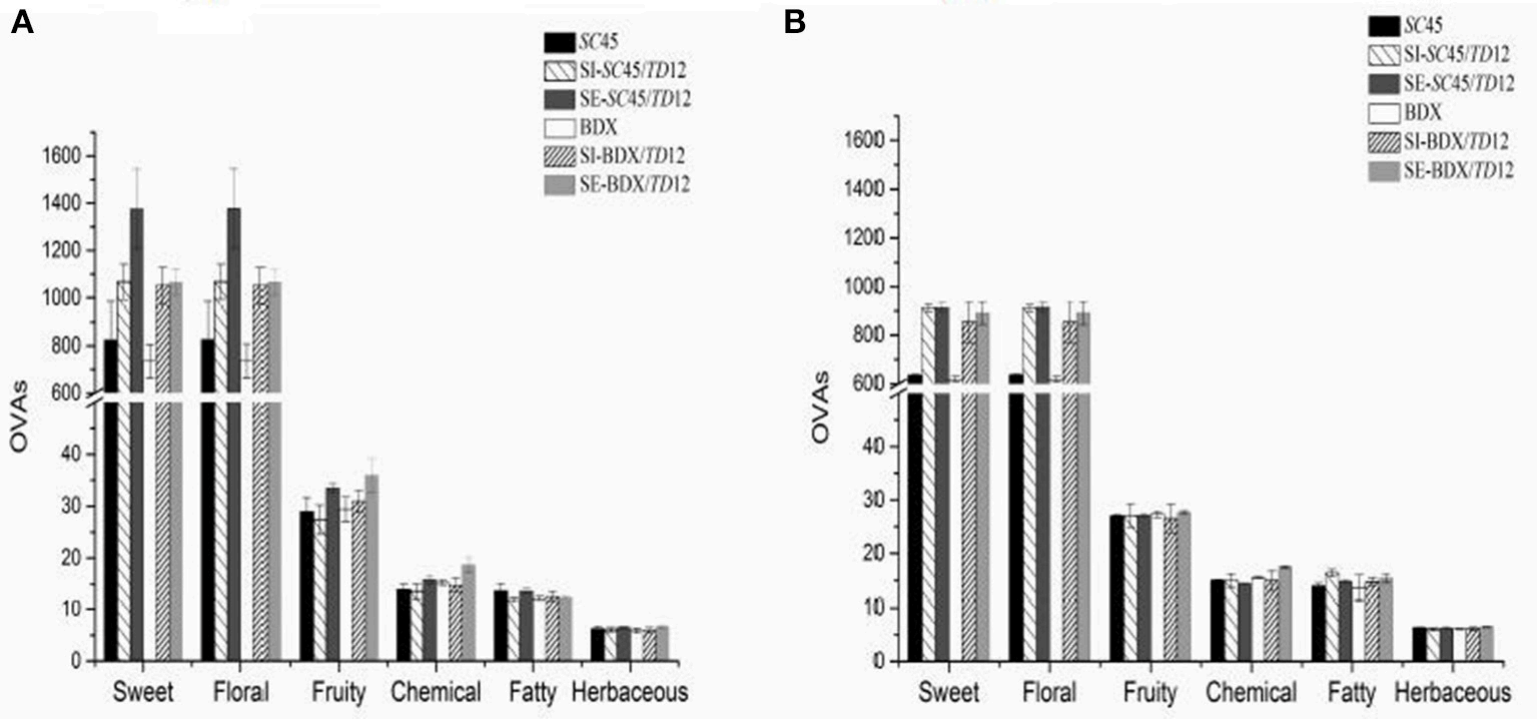
Aroma series in final wines produced by *SC*45 or BDX pure fermentation, simultaneous and sequential co-fermentation with *TD*12 after alcoholic fermentation **(A)** and malolactic fermentation **(B)**, respectively. Error bars represent standard deviations. Aroma compounds are used and calculated for floral series: 1-octanol, phenylethyl alcohol, citronellol, phenylacetaldehyde; for fruity series: 1-octanol, ethyl butanoate, isoamyl acetate, ethyl hexanoate, ethyl decanoate, ethyl acetate, geraniol; for sweet series: phenylethyl alcohol, phenylacetaldehyde; for herbaceous series: 3-methyl-1-pentanol, 1-hexanol, (Z)-3-hexen-1-ol, geraniol, decanal; for chemical series; 3-methyl-1-butanol, 3-methyl-1-pentanol; 1-octen-3-ol, ethyl acetate, 4-ethyl-phenol; for fatty series: (Z)-3-hexen-1-ol, hexanoic acid, octanomic acid, decanoic acid, 4-ethyl guaiacol.

An aromatic series could be defined as a group of volatile compounds with similar odor descriptors (Wu et al., 2011; Duan et al., 2015; Liu et al., 2016). To better understand the influence of mixed fermentation on the wine aroma profile and the contribution of different volatile compounds to the olfactory impression of wine, an aromatic series was established by combination of OAVs of a group of volatiles with similar odor descriptions (Peinado et al., 2006; Wu et al., 2011; Duan et al., 2015; Liu et al., 2016). In this research, twenty-two aroma compounds showed an OAV above 0.1 (Table 3). According to previous researches (Buettner et al., 2003; Peinado et al., 2006; Yang et al., 2008; Zhang et al., 2011; Duan et al., 2015; Liu et al., 2016), these compounds are associated with the notes “banana,” “green apple,” “citrus” “sweet,” “pear,” “pineapple,” “lemon” “roses,” “fruity,” “fatty,” “rancid,” “nail polish,” “alcohol,” “balsamic,” “floral,” and “green.” Six aromatic series of volatile compounds were finally obtained, including fruity, floral, sweet, herbaceous, chemical and fatty (Figure 3, Table S3). Of these, the floral and sweet series were prominent, followed by the fruity, chemical, fatty and herbaceous series. Sequential fermentations achieved the highest sweet, floral and fruity series due to producing more phenylethyl alcohol, ethyl butanoate, isoamyl acetate, ethyl hexanoate, ethyl decanoate, and phenylacetaldehyde, in which *SC*45/*TD*12 had higher value of sweet and floral series, while BDX/*TD*12 possessed higher fruity quality. After the malolactic fermentation, the values of sweet and floral attribution in SE wine were slightly decreased; in addition, the differences in aromatic quality between multi-cultures of *SC*45 and BDX disappeared. These results implied that the desired aromatic quality of wine generated by mixed alcoholic fermentation can be attenuated by malolactic fermentation.

## Discussion

In recent years, the use of non-*Saccharomyces* yeasts co-inoculated with *Saccharomyces cerevisiae* is a popular strategy to improve the diversity and quality of wine aroma. This application has stimulated the interest to explore the enological characteristics of non-*Saccharomyces* yeasts (species and strain). In this study, the effect of *T. delbrueckii* inoculated with two *S. cerevisiae* strains (indigenous icewine strain *SC*45 and commercial strain BDX) on the red wine aroma quality were investigate, respectively, with the aim to evaluate whether multi-culture of specific *T. delbrueckii* with different *S. cerevisiae* strains with distinct enological traits could also produce the wines with diversified aromatic quality. For achieving this goal, *S. cerevisiae SC*45 isolated from icewine spontaneous fermentation were used and compared with commercial wine yeast BDX, which is usually used to ferment table red wine. The results of pure fermentation showed that *SC*45 had distinct aromatic traits compared to BDX, such as producing higher concentrations of higher alcohols, fatty acids and ethyl esters, suggesting that *SC*45 has a good potential application in industrial wine production. As expected, multi-culture of *T. delbrueckii* with *SC*45 or BDX achieved distinct profiles of aromatic compounds. For example, SE-*SC*45/*TD*12 produced higher concentration of higher alcohol and aldehydes, and SI-*SC*45/*TD*12 generated more fatty acids ethyl esters with low level of fatty acid, while SE-BDX/*TD*12 obtained higher amount of C_6_ alcohol and acetate esters.

SE-*SC*45/*TD*12 generated the highest amount of higher alcohol, followed by SE-BDX/*TD*12, SI-*SC*45/*TD*12 and SE-BDX/*TD*12, which was corresponding to the data that pure *SC*45 produced higher concentration of high alcohol than BDX. Actually, the promotion of multi-culture of *T. delbrueckii* and *S. cerevisiae* to higher alcohol formation, especially phenylethyl alcohol, had been confirmed by several authors (Comitini et al., 2011; Azzolini et al., 2012; Sadoudi et al., 2012), although the inconsistent data was also reported (Renault et al., 2009), which were usually ascribed to the differences of *T. delbrueckii* strains used (Azzolini et al., 2015). Our results indicated that besides *T. delbrueckii* strain diversity, *S. cerevisiae* strain is also an important factor that determining higher alcohols formations in mixed fermentation. With regard to esters production, BDX can produce more acetate esters than that of *SC*45, which was still evidenced in sequential fermentation (SE-BDX/*TD*12), especially the content of isoamyl acetate (banana note). However, this promotion was not observed in the mixed fermentation of *SC*45/*TD*12. According to previous literature, *T. delbrueckii* impacting isoamyl acetate formation is strain-dependent because the increment and reduction of isoamyl acetate by mixed fermentation of *T. delbrueckii* and *S. cerevisiae* were observed by Renault et al. (2015) and Azzolini et al. (2015), respectively. Our results confirmed the positive effect of multi-culture of *T. delbrueckii* and *S. cerevisiae* on the formation of acetate esters, but also suggested that this function is not only dependent on *T. delbrueckii* strain, but also on *S. cerevisiae* strain paired inoculation. It should be mentioned that our data was different with the results of Renault et al. (2015), who proposed that interaction of *T. delbrueckii* and *S. cerevisiae* is prerequisite of synthesizing higher level of esters in mixed fermentation although the individual strain has the low ability of forming esters. But these data were consistent with the observations of Taillandier et al. (2014) and Belda et al. (2017), they believed that individual *T. delbrueckii* or *S. cerevisiae* possessing the higher ability to synthesize esters is the precondition of achieving higher contents of esters in mixed fermentation. The potential of reducing ethanol by mixed fermentation of *T. delbrueckii* or *S. cerevisiae* were also confirmed in this study. However, the reduction levels varied considerably among different fermentations (0.05%–0.82%), which largely depended on *S. cerevisiae* strain and inoculation method. This might be due to the differences of cell growths and metabolism of *T. delbrueckii* in mixed fermentation with different *S. cerevisiae* strain, because sugar can also be used for increasing the biomass of *T. delbrueckii*, or produce alternative compounds to ethanol, such as glycerol, lactic acid or malic acid in multi-culture of *T. delbrueckii* and *S. cerevisiae* (Bely et al., 2008; Belda et al., 2015). The detailed mechanism needed to be further investigated.

Our data also showed that the aromatic profiles of wines fermented by different *S. cerevisiae* strains and inoculation methods (SI and SE) were further changed after malolactic fermentation. For example, the formation of esters was attenuated, while the productions of fatty acids, terpenes and volatile phenols were enhanced after malolactic fermentation compared to after alcoholic fermentation. This implied that the differences of nutrition status in wines after alcoholic fermentation could further influence the formation of aromatic compounds in malolactic fermentation. These results collectively suggested that the formations of aromatic compounds during mixed fermentation are very complicated, which is not only dependent on inoculated yeast species and strains (non-*Saccharomyces* and *S. cerevisiae*), but also on inoculation methods, nutrition composition of must and the wine after alcoholic fermentation. Thus, it is difficult to determine and predict the aromatic impact of individual yeast species on final wine during mixed fermentation. For solving this problem, it is essential to investigate the transcriptional and metabolic response of individual yeast strains throughout wine fermentation, which can help us to rationally manipulate the formation of individual aroma compound. Our results also confirmed that sequential inoculation of *S. cerevisiae* is a better inoculation method due to generating more desired aroma compounds compared to simultaneous inoculation. This might be due to the truth that non-*Saccharomyces* strain in sequential fermentation can persist and survive longer time than that in simultaneous fermentation.

It should be emphasized that Cabernet Sauvignon is major grape variety for red wine production in China, especially in Xinjiang and Hebei wine regions. The results obtained in this study could provide an alternative way to increase the diversity of aroma profiles of Cabernet Sauvignon wine and meet the growing requirement of wine consumers for diversified aromatic quality. To further verify this conclusion, more work need to be done with other grape varieties and different vintages, and in large-scale fermenter. These experiments will be done in our future work. Considering the truth that the changes in must composition and nutrition concentration, which could be achieved by different grape variety and vintages, can result in a significant influence on the growth and metabolism of wine yeast, and alter the formation profiles of aroma compounds (Bell and Henschke, 2005), the similar results could be expected.

In sum, the present results indicated that multi-culture of *T. delbrueckii* with different *S. cerevisiae* strains possessing distinct aromatic characteristic could generate desired and diversified aromatic profiles of final wine. Compared to simultaneous inoculation, sequential inoculation is a better fermentation method because it can produce more esters, phenylethyl alcohol and phenylacetaldehyde, and intensify the fruity, flowery, and sweet characteristic of final wine. The aromatic composition after malolactic fermentation was still influenced by inoculated *S. cerevisiae* strain and inoculation methods during alcoholic fermentation. Thus, the choice of *S. cerevisiae* strains in mixed fermentation is also important for determining the aromatic quality of wines. Our results suggested that using *S. cerevisiae* strain with distinct enological traits co-fermented with one specific non-*Saccharomyces* strain is a potential way to improve the aromatic diversity and quality of wine products, which could provide an alternative way to meet the requirement of wine consumers for diversified aromatic quality.

## Author contributions

Designed the experiments: C-QD and G-LY. Conducted the experiments: YL and B-QZ. Analyzed the experimental data: B-QZ, YL, and G-LY. Wrote the paper: G-LY and B-QZ.

### Conflict of interest statement

The authors declare that the research was conducted in the absence of any commercial or financial relationships that could be construed as a potential conflict of interest. The reviewer RT and handling Editor declared their shared affiliation.
