# Supplementary material for: Use of Torulaspora delbrueckii Co-fermentation With Two Saccharomyces cerevisiae Strains With Different Aromatic Characteristic to Improve the Diversity of Red Wine Aroma Profile
Source: Front Microbiol. 2018 Apr 5;9:606. doi: 10.3389/fmicb.2018.00606 (PMC5895779; doi:10.3389/fmicb.2018.00606)
Supplement: Supplementary file 1 [file Table1.DOCX]

Supplementary Material

**Use of *Torulaspora delbrueckii* co-fermentation with** **two *Saccharomyces cerevisiae* strains with different aromatic characteristic to improve the diversity of red wine aroma profile**

Bo-Qin Zhang^1,2^, Yu Luan^1,2^, Chang-Qing Duan^1,2^, Guo-Liang Yan^1,2*^

*** *Author for correspondence (Tel: +86-10-62737039; Fax: +86-10-62738658; E-mail:*** [***glyan@cau.edu.cn***](mailto:glyan@cau.edu.cn)***)***

# 1. Supplementary Tables

**TABLE S1 | The ratio of nitrogenous compounds of malolactic fermentation *vs* alcoholic fermentation（%）**

| **Nitrogenous compounds** | ***SC*45** | **SI-*SC*45/*TD*12** | **SE-*SC*45/*TD*12** | **BDX** | **SI-BDX/*TD*12** | **SE-BDX*/TD*12** |
| --- | --- | --- | --- | --- | --- | --- |
| Asp | 128.28±2.9 | 54.26±1.11 | 136.04±14.84 | 198.69±3.49 | 88.31±3.84 | 128.4±1.38 |
| Glu | 78.5±1.95 | 34.26±1.01 | 107.96±1.99 | 119.91±3.44 | 60.34±40.71 | 120.04±1.62 |
| Ser | 41.81±0.79 | 16.09±2.81 | 23.53±2.08 | 17.6±1.54 | -4.79±0.26 | 17.97±1.57 |
| Asn | -42.87±0.81 | -2.57±24.16 | 36.58±55.6 | -24.07±2.36 | -40.49±0.20 | -2.95±2.21 |
| Gln | 39.86±2.63 | 6.53±9.85 | 13.14±0.78 | 4.17±0.66 | -10.75±2.17 | 6.88±5.57 |
| His | 45.82±0.52 | 59.52±2.77 | 98.76±3.10 | 10.05±1.59 | 101.49±1.86 | 160.78±3.5 |
| Gly | 56.52±0.33 | 43.38±1.84 | 45.12±1.01 | 58.23±0.95 | 33.59±0.26 | 50.66±0.13 |
| Thr | 43.26±3.31 | 81.36±84.55 | 30.58±49.14 | 133.63±2.69 | 11.71±0.49 | 45.93±2.91 |
| β-Ala | 88.17±0.12 | 36.23±3.04 | 13.81±1.38 | 92.15±3.22 | 22.44±0.80 | 31.20±3.50 |
| Arg | -27.63±5.07 | -56.85±0.13 | -46.08±2.60 | -42.93±0.91 | -32.95±0.65 | -28.99±0.55 |
| Ala | 210.75±14.02 | 52.89±2.83 | 154.22±14.54 | 212.63±5.38 | 88.49±1.26 | 137.02±1.36 |
| GABA | 161.19±1.68 | 60.46±2.15 | 69.54±2.44 | 145.25±4.64 | 79.62±10.50 | 121.66±1.27 |
| Pro | 7.80±0.34 | 9.52±1.86 | 11.53±0.57 | 6.02±0.36 | -0.51±0.57 | 9.69±0.07 |
| NH4+ | 241.94±27.45 | 226.59±5.74 | 432.85±55.8 | 231.43±20.30 | 193.66±2.80 | 355.53±36.60 |
| Tyr | 30.01±2.95 | 47.33±0.41 | -24.89±0.99 | 36.56±2.00 | 14.93±2.55 | 48.74±0.42 |
| Val | 57.53±1.64 | 43.38±1.32 | 40.50±1.82 | 68.70±1.34 | 22.80±1.35 | 49.13±0.52 |
| Met | 112.36±5.72 | 116.97±42.24 | 20.22±0.36 | 33.98±0.35 | -18.21±1.12 | -17.01±3.55 |
| Cys | -9.79±29.5 | 34.37±1.28 | 32.00±33.68 | 148.66±8.56 | 74.47±24.26 | 173.72±14.7 |
| Ile | -19.78±1.85 | -24.32±1.74 | -41.00±2.05 | -24.09±9.32 | -38.79±8.52 | -23.26±7.24 |
| Leu | 64.71±2.32 | 33.78±0.67 | 38.22±0.89 | 45.88±4.23 | 31.59±13.58 | 74.13±13.95 |
| Trp | 4.81±21.27 | 20.14±1.19 | 18.83±1.24 | 14.61±1.05 | 3.30±1.74 | 16.36±2.31 |
| Phe | 7.82±1.42 | 35.8±0.88 | 10.28±1.47 | 14.11±2.58 | 2.94±0.15 | 23.61±1.28 |
| Orn | 337.52±46.51 | 383.97±3.53 | 566.13±10.08 | 424.66±7.05 | 381.95±4.51 | 968.75±8.75 |
| Lyr | -23.79±57.52 | -66.05±1.85 | -70.54±1.49 | -70.29±2.06 | -30.46±5.30 | -32.72±1.85 |
| YAN | 55.79±0.17 | 31.36±4.41 | 50.75±0.35 | 44.86±0.22 | 27.15±1.82 | 65.87±0.51 |

*SC45: S. cerevisiae SC45 pure fermentation; SI-SC45/TD12: Simultaneous inoculation of SC45 and TD12; SE-SC45/TD12: Sequential inoculation of TD12 followed by SC45 after 2 days; BDX*: *S. cerevisiae BDX pure fermentation; SI-BDX/TD12: Simultaneous inoculation of BDX and TD12; SE-BDX/TD12: Sequential inoculation of TD12 followed by BDX after 2 days.*

*All data are shown as mean values ± standard deviations.*

**TABLE S2 | Volatile composition（µg/L）of experimental wines after malolactic fermentation**

| **Aroma compounds** | ***SC*45** | **SI-*SC*45/*TD*12** | **SE-*SC*45/*TD*12** | **BDX** | **SI-BDX/*TD*12** | **SE-BDX/*TD*12** |
| --- | --- | --- | --- | --- | --- | --- |
| 1-Hexanol | **5360.69±116.68^ab^** | **5075.14±236.27^b^** | **5247.21±110.71^b^** | **5139.97±105.78^b^** | **5069.48±118.18^b^** | **5612.29±106.82^a^** |
| (E)-3-Hexen-1-ol | 97.67±7.48^a^ | 76.85±13.84^b^ | 97.56±3.54^a^ | 96.27±8.07^ab^ | 83.79±4.17^ab^ | 93.14±2.29^ab^ |
| (Z)-3-Hexen-1-ol | 139.53±0.59^d^ | 146.84±2.04^c^ | 195.01±3.88^a^ | 141.79±5.03^cd^ | 140.97±1.64^cd^ | 156.5±0.79^b^ |
| **Total C_6_ alcohols** | 5597.88±124.75^b^ | 5298.79±252.14^d^ | 5539.78±118.13^b^ | 5378.03±118.89^c^ | 5294.24±123.95^d^ | 5861.94±109.91^a^ |
| 3-Methyl-1-butanol | **161510.4±2703.1^bc^** | **190335.1±9860.4^b^** | **209752.1±1246.5^ab^** | **154751.9±293.8^c^** | **191025.4±19539.6^b^** | **230475.3±2104.1^a^** |
| 3-Methyl-1-pentanol | 230.53±1.51^a^ | 233.82±19.24^a^ | 212.16±0.43^a^ | 229.74±0.95^a^ | 237.07±23.16^a^ | 218.08±3.48^a^ |
| 4-Methyl-1-pentanol | 5.15±0.95^a^ | 5.39±1.45^a^ | 5.66±0.13^a^ | 5.56±0.06^a^ | 5.94±0.87^a^ | 5.99±0.16^a^ |
| 2-Octanol | 1.6±0.03^a^ | 1.51±0.39^a^ | 1.93±0.65^a^ | 1.78±0.31^a^ | 1.54±0.01^a^ | 1.65±0.01^a^ |
| 1-Octen-3-ol | 13.64±0.52^ab^ | 11.74±0.65^c^ | 12.05±0.06^c^ | 14.41±0.74^a^ | 12.24±0.92^bc^ | 12.3±0.06^bc^ |
| 2-Ethyl-1-hexanol | 3.87±0.14^ab^ | 3.83±0.84^ab^ | 3.41±0.12^c^ | 4.07±0.18^a^ | 3.64±0.65^b^ | 3.39±0.08^c^ |
| 2-Nonanol | 3.57±0.14^b^ | 2.52±0.08^d^ | 3.19±0.16^c^ | 3.33±0.01^bc^ | 2.44±0.18^d^ | 4.02±0.24^a^ |
| 1-Octanol | 229.98±8.92^c^ | 269.21±40.19^bc^ | 302.96±6.25^ab^ | 246.68±6.32^c^ | 259.01±31.66^bc^ | 339.49±5.04^a^ |
| (6Z)-Nonen-1-ol | 9.93±0.44^a^ | 7.87±0.34^b^ | 7.64±0.21^b^ | 9.24±0.54^a^ | 8.11±0.28^b^ | 9.96±0.25^a^ |
| 1-Decanol | 5.97±0.19^d^ | 7.31±0.55^c^ | 12.73±0.63^b^ | 5.7±0.14^d^ | 7.08±0.57^c^ | 15.25±0.09^a^ |
| Benzyl alcohol | 419.57±6.99^c^ | 507.81±58.22^a^ | 210.95±254.53^d^ | 477.71±133.12^b^ | 424.87±18.15^c^ | 439.67±22.87^bc^ |
| Phenylethyl alcohol | **124648.4±4220.7^c^** | **247570.7±43425.1^a^** | **211156.8±10537.8^ab^** | **145313.2±56068.4^bc^** | **206029.8±24881.6^ab^** | **208468.8±18757.8^ab^** |
| **Total of higher alcohols** | 292680.5±7065.2^c^ | 444255.5±53505.9^a^ | 427221.5±11647.8^ab^ | 306441.3±56397.8^c^ | 403311.3±34499.4^b^ | 445855.8±20772.4^a^ |
| Hexanoic acid | **1656.09±249.81^b^** | **1837.29±125.99^a^** | **1476.47±58.46^c^** | **1592.54±203.28^b^** | **1621.57±81.46^b^** | **1628.43±133.51^b^** |
| Octanoic acid | **2103.79±7.81^d^** | **2955.04±141.04^a^** | **2332.58±29.32^c^** | **2030.75±75.69^e^** | **2451.79±275.81^b^** | **2368.79±186.43^bc^** |
| n-Decanoic acid | 219.26±1.65^bc^ | 240.15±3.15^ab^ | 243.71±5.91^ab^ | 203.53±22.07^c^ | 231.95±6.32^ab^ | 252.54±5.74^a^ |
| **Total of fatty acids** | 3979.14±240.35^a^ | 5032.49±263.89^a^ | 4052.76±93.68^a^ | 3826.79±301.04^a^ | 4305.31±363.6^a^ | 4249.76±325.68^a^ |
| Isoamyl acetate | **1108.99±14^a^** | **1168.9±131.23^a^** | **1269.18±16.81^a^** | **1075.56±39.57^a^** | **1163.54±171.61^a^** | **1042.67±13.45^a^** |
| Hexyl acetate | 7.48±0.18^a^ | 5.3±0.27^c^ | 6.28±0.12^b^ | 7.14±0.68^ab^ | 4.84±0.44^c^ | 1.62±0.17^d^ |
| Phenethyl acetate | 39.48±1.08^c^ | 49.82±2.76^b^ | 54.96±1.08^a^ | 39.16±2.01^c^ | 47.21±3.24^b^ | 48.03±0.77^b^ |
| Ethyl acetate | **62647.55±184.82^ab^** | **56034.3±6530.11^bc^** | **46576.16±351.31^c^** | **67557.57±479.42^a^** | **54991.7±8007.83^bc^** | **64704.88±980.68^ab^** |
| **Total of acetate esters** | 63803.5±199.71^ab^ | 57258.32±6658.3^bc^ | 47906.58±367.16^c^ | 68679.43±517.65^a^ | 56207.28±8176.67^bc^ | 65797.2±993.47^ab^ |
| Ethyl dodecanate | 34.85±1.12^b^ | 34.9±1.05^b^ | 40.61±1.25^a^ | 34.12±0.5^b^ | 35.23±1.34^b^ | 43.03±0.31^a^ |
| Ethyl butanoate | **437.32±4.28^c^** | **478.27±15.86^ab^** | **427.64±1.12^cd^** | **483.68±1.45^a^** | **413.65±6.66^d^** | **463.77±3.12^b^** |
| Ethyl hexanoate | **354.42±4.25^ab^** | **364.37±11.71^ab^** | **389.27±1.68^a^** | **343.21±17.38^b^** | **353.54±27.87^ab^** | **339.35±4.35^b^** |
| Ethyl heptanoate | 0.55±0.02^b^ | 0.55±0.01^b^ | 0.67±0.05^a^ | 0.49±0.02^b^ | 0.52±0.03^b^ | 0.5±0.01^b^ |
| Ethyl lactate | 77735.71±597.37^ab^ | 100048.49±25827.71^ab^ | 104152.06±2296.14^a^ | 73707.77±2720.46^b^ | 90244.1±9963.69^ab^ | 95526.1±422.48^ab^ |
| Ethyl octanoate | 49.98±0.17^c^ | 60.45±4.34^ab^ | 67.97±2.22^a^ | 49.79±0.85^c^ | 56.67±5.76^bc^ | 62.85±0.35^ab^ |
| Ethyl nonanoate | 1.25±0.01^d^ | 1.33±0.03^c^ | 1.57±0.03^a^ | 1.12±0.01^e^ | 1.45±0.03^b^ | 1.29±0.01^cd^ |
| Ethyl decanoate | **879.72±0.28^e^** | **912.59±8.12^c^** | **984.65±7.38^b^** | **867.02±0.95^f^** | **899.72±3.41^d^** | **1007.04±2.99^a^** |
| **Total of ethyl esters** | 79494.54±605.36^ab^ | 101905,91±25790.96^ab^ | 106074.34±2305.29^a^ | 75488.01±2700.85^b^ | 92008.99±9936^ab^ | 97445.82±433.2^ab^ |
| Isoamyl octanoate | 4.34±0.01^d^ | 5.63±0.72^c^ | 6.13±0.12^a^ | 4.23±0.05^d^ | 5.19±0.34^bc^ | 7.02±0.01^b^ |
| Diethyl succinate | 445.87±12.89^c^ | 1028.15±24.42^a^ | 821.57±40.24^b^ | 697.91±76.37^c^ | 927.59±19.76^ab^ | 858.01±43.61^b^ |
| Methyl octanoate | 1.17±0.12^c^ | 1.33±0.15^b^ | 1.65±0.04^a^ | 1.12±0.02^c^ | 1.25±0.12^bc^ | 1.38±0.01^b^ |
| Isopentyl hexanoate | 3.55±0.04^d^ | 4.13±0.26^bc^ | 4.54±0.07^ab^ | 3.46±0.01^d^ | 4.02±0.37^c^ | 4.68±0.09^a^ |
| **Total of other esters** | 455.67±12.86^d^ | 1040.09±23.47^a^ | 834.64±40.45^b^ | 707.41±76.45^c^ | 938.87±20.43^ab^ | 871.82±43.53^b^ |
| Decanal | 2.59±0.24^b^ | 2.59±0.27^b^ | 2.29±0.56^c^ | 2.48±0.12^bc^ | 3.85±1.68^a^ | 2.1±0.05^d^ |
| Benzaldehyde | 14.63±0.29^ab^ | 14.47±0.78^b^ | 13.6±0.44^c^ | 14.78±1.28^ab^ | 13.71±0.66^c^ | 15.36±0.13^a^ |
| Phenylacetaldehyde | **626.89±3.44^c^** | **996.92±141.65^a^** | **897.77±20.25^ab^** | **695.59±118.93^bc^** | **838.78±84.56^abc^** | **875.93±46.97^ab^** |
| **Total of aldehydes** | 644.1±2.91^c^ | 1013.98±142.16^a^ | 913.67±19.25^ab^ | 712.85±120.33^bc^ | 856.34±86.9^abc^ | 893.38±47.15^ab^ |
| Linalool | 1.54±0.04^b^ | 1.68±0.09^ab^ | 1.76±0.07^a^ | 1.61±0.07^b^ | 1.67±0.05^ab^ | 1.83±0.03^a^ |
| Citronellol | 10.82±0.09^c^ | 12.98±0.67^ab^ | 13.04±1.07^a^ | 11.53±0.29^bc^ | 12.88±0.38^ab^ | 13.9±0.47^a^ |
| Geraniol | **20.43±0.08^a^** | **20.99±0.08^a^** | **20.45±0.07^a^** | **20.43±0.47^a^** | **20.44±0.24^a^** | **20.53±0.24^a^** |
| Farnesol | 25.08±0.19^b^ | 29.92±0.38^a^ | 24.54±0.98^b^ | 28.28±2.83^a^ | 29.05±0.84^a^ | 24.83±0.14^b^ |
| **Total of terpenes** | 94.58±1.42^b^ | 103.16±4.14^a^ | 108.42±3.09^a^ | 101.45±4.26^ab^ | 100.29±4.03^ab^ | 104.81±0.91^a^ |
| Phenol | 65.95±0.94^b^ | 80.45±4.7^a^ | 78.47±0.87^ab^ | 73.39±11.44^ab^ | 70.02±1.93^ab^ | 75.85±1.82^ab^ |
| 4-Ethyl guaiacol | **32.06±0.08^c^** | **36.73±2.22^bc^** | **40.07±1.05^ab^** | **35.9±3.75^bc^** | **33.86±2.93^bc^** | **43.54±3.19^a^** |
| 4-Ethyl-phenol | 46.53±0.97^c^ | 54.45±3.12^ab^ | 60.31±1.85^a^ | 59.45±0.49^a^ | 48.77±4.17^bc^ | 58.83±3.85^a^ |
| **Total of volatile phenols** | 144.49±1.99^c^ | 171.63±0.64^ab^ | 178.85±3.69^a^ | 168.74±14.66^ab^ | 152.65±9.04^bc^ | 178.23±8.81^a^ |

*SC45: S. cerevisiae SC45 pure fermentation; SI-SC45/TD12: Simultaneous inoculation of SC45 and TD12; SE-SC45/TD12: Sequential inoculation of TD12 followed by SC45 after 2 days; BDX*: *S. cerevisiae BDX pure fermentation; SI-BDX/TD12: Simultaneous inoculation of BDX and TD12; SE-BDX/TD12: Sequential inoculation of TD12 followed by BDX after 2 days.*

*The aroma compounds (OVA>1) were highlighted and underlined, other volatile compounds (OVA>0.1) were underlined. Values are given as mean ± standard deviation of two biological replicates and three detection runs. Data with different superscript letters (a, b, c, d, e, f) within each column are different according to Duncan tests (0.05%).*

**TABLE S3 | Volatile compounds identified (OVA>0.1) and their aroma parameters**

| **Aroma compounds** | **Odor value** | **Odor description** | **Aromatic series** |
| --- | --- | --- | --- |
| 3-Methyl-1-butanol | 30000 | alcohol, nail polish | Chemical |
| 3-Methyl-1-pentanol | 500 | pungent, solvent, green | Chemical，Herbaceous |
| 1-Hexanol | 1100 | herbaceous, grass, woody | Herbaceous |
| (Z)-3-Hexen-1-ol | 1000 | herbaceous, green, bitter, fatty | Herbaceous，Fatty |
| 1-Octen-3-ol | 20 | mushroom | Chemical |
| 1-Octanol | 800 | jasmine, lemon | Floral，Fruity |
| Phenylethyl alcohol | 14000 | pollen, roses | Floral，Sweet |
| Hexanoic acid | 420 | fatty, cheese, rancid | Fatty |
| Octanoic acid | 500 | fatty, rancid, harsh, cheese | Fatty |
| Decanoic acid | 1000 | rancid, cheese | Fatty |
| Ethyl butanoate | 400 | banana, pineapple, strawberry | Fruity |
| Isoamyl acetate | 160 | banana | Fruity |
| Ethyl hexanoate | 80 | fruity, green apple, banana | Fruity |
| Ethyl decanoate | 200 | fruity, pleasant | Fruity |
| Ethyl acetate | 7500 | pineapple, varnish, balsamic | Fruity，Chemical |
| Citronellol | 100 | rose | Floral |
| Geraniol | 20 | vitric, geranium | Herbaceous，Fruity |
| Decanal | 10 | green, fresh | Herbaceous |
| Phenylacetaldehyde | 1 | floral，honey | Floral，Sweet |
| 4-Ethyl guaiacol | 33 | smoky | Fatty |
| 4-Ethyl-phenol | 450 | phenolic, leather | Chemical |

**Table S4 | CAS, RI, Quantitative Ion, Quantitative standards, Calibration curves and R^2^ for quantification of volatile compounds in this study**

| Aroma compounds | CAS | RI^a^ | Quantitative ion(m/z)^b^ | Quantitative standards | ID^c^ | Calibration curves | R^2^ |
| --- | --- | --- | --- | --- | --- | --- | --- |
| 1-Hexanol | 111273 | 1347.1 | 56 | 1-Hexanol | A | y = 1636.43x＋0.00 | 0.999 |
| (E)-3-Hexen-1-ol | 928972 | 1359.5 | 41 | (E)-3-Hexenol | A | y = 446.86x＋4.81 | 0.994 |
| (Z)-3-Hexen-1-ol | 928961 | 1381 | 67 | (Z)-3-Hexenol | A | y = 7898.50x＋35.81 | 0.99 |
| 3-Methyl-1-butanol | 123513 | 1256 | 86 | 3-Methyl-2-butenol | B | y = 48907.17x-4.52 | 0.997 |
| 3-Methyl-1-pentanol | 763326 | 1244.8 | 86 | 3-Methyl-2-butenol | B | y = 48907.17x-4.52 | 0.997 |
| 4-Methyl-1-pentanol | 626891 | 1316 | 86 | 3-Methyl-2-butenol | B | y = 48907.17x-4.52 | 0.997 |
| 2-Octanol | 123966 | 1414.2 | 45 | 2-Octanol | A | y = 875.89x＋0.09 | 0.996 |
| 1-Octen-3-ol | 3391864 | 1447.7 | 57 | 1-Octen-3-ol | A | y = 218.64x＋3.15 | 0.997 |
| 2-Ethyl-1-hexanol | 104767 | 1488 | 57 | 2-Ethyl-1-hexanol | A | y = 372.02x-0.30 | 0.976 |
| 2-Nonanol | 628999 | 1516.5 | 45 | 2-Nonanol | A | y = 1010.45x＋0.10 | 0.998 |
| 1-Octanol | 111875 | 1557.8 | 56 | 1-Octanol | A | y = 1297.17x＋1.73 | 0.959 |
| (6Z)-Nonen-1-ol | 143088 | 1662.3 | 45 | 2-Nonanol | B | y = 1010.45x＋0.10 | 0.998 |
| 1-Decanol | 112301 | 1766 | 70 | 1-Decanol | A | y = 706.34x-0.03 | 0.989 |
| Benzyl alcohol | 100516 | 1892.5 | 79 | Benzyl alcohol | A | y = 15127.68x＋141.11 | 0.956 |
| Phenylethyl alcohol | 60128 | 1927.6 | 91 | Phenylethyl Alcohol | A | y = 14927.33x＋274.98 | 0.985 |
| Hexanoic acid | 142621 | 1859 | 60 | Hexanoic acid | A | y = 9412.52x＋40.68 | 0.979 |
| Octanoic acid | 124072 | 2075 | 60 | Octanoic acid | A | y=1105.19x+89.46 | 0.992 |
| n-Decanoic acid | 334485 | 2292 | 60 | n-Decanoic acid | A | y=710.12x+73.45 | 0.981 |
| Isoamyl acetate | 123922 | 1130.5 | 43 | Isoamyl acetate | A | y = 966.18x-0.30 | 0.98 |
| Hexyl acetate | 142927 | 1270.5 | 43 | Hexyl acetate | A | y = 1612.24x-0.03 | 0.986 |
| Phenethyl acetate | 103457 | 1830 | 104 | Phenethyl acetate | A | y=95.74x+1.23 | 0.999 |
| Ethyl acetate | 141786 | 789.1 | 43 | Ethyl Acetate | A | y = 1896.30x＋0.00 | 0.999 |
| Ethyl dodecanate | 106332 | 1848 | 88 | Ethyl dodecanoate | A | y=58.72x+70.38 | 0.982 |
| Ethyl butanoate | 105544 | 1039.5 | 71 | Ethyl butanoate | A | y = 1271.59x＋0.49 | 0.999 |
| Ethyl hexanoate | 123660 | 1231.3 | 88 | Ethyl hexanoate | A | y = 1085.22x＋1.29 | 0.964 |
| Ethyl heptanoate | 106309 | 1334 | 88 | Ethyl hexanoate | B | y = 1085.22x＋1.29 | 0.964 |
| Ethyl lactate | 97643 | 1350 | 45 | Ethyl lactate | A | y=56382.02x+117.63 | 0.999 |
| Ethyl octanoate | 106321 | 1434.1 | 88 | Ethyl octanoate | A | y = 6713.74x-0.97 | 0.988 |
| Ethyl nonanoate | 123295 | 1535 | 88 | Ethyl nonanoate | A | y=17.14x+1.03 | 0.984 |
| Ethyl decanoate | 110383 | 1642 | 88 | Ethyl decanoate | A | y = 9118.92x＋0.90 | 0.984 |
| Isoamyl octanoate | 2035996 | 1659 | 70 | Isoamyl hexanoate | B | y=38.49x+14.87 | 0.996 |
| Diethyl succinate | 123251 | 1682 | 101 | Diethyl succinate | A | y=1586.48x-244.23 | 0.994 |
| Methyl octanoate | 111115 | 1390 | 74 | Methyl octanoate | A | y=22.47x+0.14 | 0.999 |
| Isopentyl hexanoate | 2198610 |  |  | - | C^d^ | - | - |
| Decanal | 112312 | 1501.9 | 43 | Decanal | A | y = 6998.38x-0.85 | 0.969 |
| Benzaldehyde | 100527 | 1536.4 | 77 | Benzaldehyde | A | y = 6053.04x＋6.01 | 0.979 |
| Phenylacetaldehyde | 122781 | 1659.1 | 91 | Benzeneacetaldehyde | A | y = 6327.32x＋72.26 | 0.968 |
| Linalool | 78706 | 1548.3 | 71 | Linalool | A | y = 512.09x＋1.37 | 0.957 |
| Citronellol | 106229 | 1770 | 41 | Citronellol | A | y=237.08x+0.19 | 0.997 |
| Farnesol | 4602840 |  |  | - | C^d^ | - | - |
| Geraniol | 106241 | 1853.8 | 69 | Geraniol | A | y = 7950.19x＋3.27 | 0.975 |
| Phenol | 108952 | 2029 | 94 | Phenol | A | y=2211.20x-0.17 | 0.999 |
| 4-Ethyl guaiacol | 90051 | 1877.6 | 109 | 4-Ethyl guaiacol | A | y = 52975.58x＋1.92 | 0.981 |
| 4-Ethyl-phenol | 123079 | 2198 | 107 | 4-Ethyl phenol | A | y=444.45x+1.44 | 0.991 |

^a^ Retention indices were calculated on HP-INNOWAX column. ^b^ Quantitative ion for peak area evaluation of volatile compounds. ^c^ Identification of volatile compounds: A, identified by mass spectrum and RI agreed with standards; B and C, identified by spectrum and RI agreed with NIST 11 MS database and literature data. ^d^ The concentration of these compounds expressed as relative areas (to 4-methyl-2-pentanol).
